# Neurodegenerative changes initiated by presynaptic dysfunction

**DOI:** 10.1186/2047-9158-2-16

**Published:** 2013-08-07

**Authors:** Toru Yasuda, Yasuto Nakata, Chi-Jing Choong, Hideki Mochizuki

**Affiliations:** 1 Department of Neurology, Osaka University Graduate School of Medicine, Suita, Osaka, Japan; 2 Present address: Department of Genetics, National Center for Child Health and Development, Setagaya-ku, Tokyo, Japan

## Abstract

α-Synucleinopathies are a subgroup of neurodegenerative diseases including dementia with Lewy bodies (DLB) and Parkinson’s disease (PD). Pathologically, these disorders can be characterized by the presence of intraneuronal aggregates composed mainly of α-synuclein (αSyn), which are called Lewy bodies and Lewy neurites. Recent report showed that more than 90% of αSyn aggregates are present in the form of very small deposits in presynaptic terminals of the affected neurons in DLB. However, the mechanisms responsible for presynaptic accumulation of abnormal αSyn remain unclear. In this article, we review recent findings on the involvement of presynaptic dysfunction in the initiation of neuronal dysfunctional changes. This review highlights that the presynaptic failure can be a potential trigger of the dying-back neuronal death in neurodegenerative diseases.

## Introduction

Neurodegenerative diseases are age-associated and progressive disorders, which detrimentally affect patients’ quality of life. Medical remedies that can fully cure the diseases are currently unavailable and invention of novel therapeutic applications is urgently required. Accordingly, it is important to identify the initial trigger(s) of the pathophysiological alterations in these diseases.

α-Synucleinopathies are a subgroup of neurodegenerative diseases including dementia with Lewy bodies (DLB), Parkinson’s disease (PD), and multiple system atrophy (MSA). Pathological hallmark of these disorders is the formation of intracellular aggregates composed mainly of α-synuclein (αSyn), which are called Lewy bodies and Lewy neurites [1-3]. Pathological examination of DLB patients has identified the presence of abnormal α-synuclein (αSyn) aggregates in the presynaptic terminals [4-6]. However, the mechanisms responsible for presynaptic accumulation of abnormal αSyn remain elusive.

### Role of αSyn in SNARE formation

αSyn is abundantly localized in the presynaptic nerve terminals [7,8]. The physiological functions of αSyn have yet to be defined, while several lines of evidence implicated this protein in the modulation of neurotransmitter release through the regulation of soluble *N*-ethylmaleimide-sensitive factor attachment protein receptor (SNARE) complex formation [9-11] and size of synaptic vesicle pool [12-15]. Vesicle-associated membrane protein-2 (VAMP-2) present in the synaptic vesicles, and syntaxin and synaptosomal-associated protein of 25 KDa (SNAP-25) in the presynaptic plasma membrane form the core SNARE complex, which regulate docking and fusion of synaptic vesicles to the presynaptic membrane [16]. A recent study showed the physical interaction of αSyn with VAMP-2 promotes SNARE assembly [10]. Cysteine-string protein-α (CSPα) also participates in SNARE assembly and mutant mice lacking CSPα displayed impaired SNARE formation and premature death, but both of these phenotypes are counteracted by transgenic expression of αSyn [9,17]. On the other hand, overexpression of αSyn with no overt toxicity inhibits neurotransmitter release, due to a defective reclustering of synaptic vesicles after endocytosis [15]. Additionally, overexpressed αSyn indirectly inhibits SNARE-mediated exocytosis by sequestering arachidonic acid, which upregulates syntaxin and enhances its engagement with SNARE complex [11]. Importantly, abnormal redistribution of SNARE proteins has been observed in human PD patients and mice overexpressing a truncated form of human αSyn, which showed decreased release of dopamine (DA) in the striatum [18]. Therefore, presynaptic SNARE dysfunction is considered an initial pathogenic event in α-synucleinopathies.

### Accumulation of α-synuclein triggered by presynaptic dysfunction

**Figure 1:**
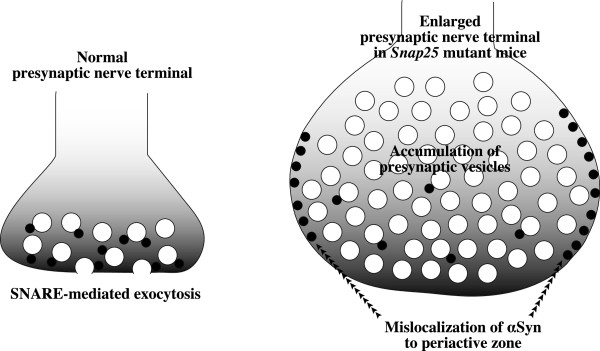
**A hypothetical diagram showing enlarged presynaptic nerve terminals in SNAP-25 mutant mice.** Normal presynaptic nerve terminals with uniform distribution of vesicles were observed in wild type mice while abnormally enlarged presynaptic nerve terminals with condensed synaptic vesicles and predominant localization of αSyn proteins in the periactive zones were found in SNAP-25 mutant mice, suggesting that SNARE dysfunction leads to presynaptic accumulation of endogenous αSyn and perturbations to the finely-tuned balance between exocytosis and endocytosis.

In our recent study, we investigated the effects of SNARE dysfunction on endogenous αSyn using *Snap25*^*S187A/S187A*^ mutant mice [19]. These mice have homozygous knock-in gene encoding unphosphorylatable S187A-substituted SNAP-25. *Snap25*^*S187A/S187A*^ mutant mice present a concomitant reduction of neurotransmitter release, including serotonin and DA, from the amygdala, and develop convulsive seizures and anxiety-related behavior in general activity and light-and-dark preference tests [20]. We found that the mutant mice displayed a significant age-dependent change in the distribution of αSyn and its Ser^129^-phosphorylated form in abnormally hypertrophied glutamatergic nerve terminals in the striatum. Electron microscopic analysis revealed the atypically condensed synaptic vesicles with concomitant mislocalization of αSyn protein to the periactive zone in the glutamatergic nerve terminals (Figure 1). However, the *Snap25*^*S187A/S187A*^ mutant mice harbored no abnormalities in the nigrostriatal dopaminergic neurons [19]. Our results suggest that SNARE dysfunction is the initial trigger of mislocalization and accumulation of αSyn, and probably underlies the pathomechanism of α-synucleinopathies.

### Effect of SNAP-25 dysfunction

Previous studies using neural preparations showed that the neurotransmitter release is regulated by protein kinase C, which phosphorylates Ser^187^ residue in SNAP-25, augmenting exocytosis of synaptic vesicles [21,22]. Patch-clamp analysis showed chromaffin cells that overexpressed the S187A mutant form of SNAP-25 had impaired rate of presynaptic vesicle pool refilling [23]. Recently, we reported that *Snap25*^*S187A/S187A*^ mice showed reduced DA and serotonin release in amygdala [20]. In human DLB brains, more than 90% of αSyn aggregates are located in the presynaptic terminals in the form of small deposits [4-6]. This is consistent with our findings of abnormal accumulation of αSyn in presynapses [19], suggesting that this process is the initial pathological event in DLB, eventually leading to the death and degeneration of neuronal cells [24]. Another finding that lends support to the role of αSyn aggregates in the presynaptic terminals in DLB is the lack of histopathological changes in the dopaminergic terminals in our study [19].

### Role of αSyn in nerve terminals

In experiments on glutamate release conducted in hippocampal slices prepared from αSyn knockout mice [25], paired-pulse facilitation was significantly weaker, and high-frequency-induced long-term potentiation and frequency facilitation were not observed. These findings suggest that αSyn contributes to mobilization of glutamate-containing vesicles from the reserve pool [25]. αSyn may act as a positive regulator of neurotransmitter release at presynaptic terminals. Therefore, presynaptic accumulation of αSyn observed in our *Snap25*^*S187A/S187A*^ mice might reflect a compensatory response to a possible SNARE dysfunction-related chronic shortage of neurotransmitter release in the VGLUT1-positive nerve terminals [19].

### Relation between glutamatergic and dopaminergic nerve terminals in the striatum

In the striatum, the medium spiny neurons, which constitute more than 90% of all striatal neurons, receive input from glutamatergic axons that contact the spine head and dopaminergic axons that synapse with the dendritic spine neck. DA released from dopaminergic axons regulates the release of glutamate via D_2_-like receptors on the corticostriatal nerve terminals [26,27]. We found no significant changes in the striatal tissue levels of DA and its metabolites in *Snap25*^*S187A/S187A*^ mice. These findings confirmed the results reported in our previous study using the same mouse model, in which the microdialysis analysis revealed marked reduction of DA release from the amygdala [20]. In another *in vitro* study using PC12 cells, phosphorylation of SNAP-25 at S187 potentiated calcium-dependent DA release and recruitment of synaptic vesicles containing DA [28-30]. These observations suggest decreased striatal DA release in *Snap25*^*S187A/S187A*^ mice, resulting in increased demand for neurotransmitter release at glutamatergic nerve terminals. Thus, presynaptic accumulation of αSyn might reflect a possible compensatory response to low DA inhibitory control over cortical glutamatergic drive.

### Correlation of VAMP-2 with αSyn

Increased expression of VAMP-2 protein accompanied increased αSyn expression in the striatum of *Snap25*^*S187A/S187A*^ mice [19]. Binding of the carboxy terminus of αSyn to the amino terminus of VAMP-2 primes subsequent SNARE complex assembly [31]. Therefore, the increased VAMP-2 level might also reflect a compensatory response to the impaired synaptic vesicle release by enhancing SNARE complex formation in concert with increased αSyn.

### Pathological changes in glutamatergic nerve terminals

Presynaptic neurotransmitter release is mediated by the synaptic vesicle cycle, consisting of exocytosis followed by endocytosis and recycling. Exocytosis incorporates synaptic vesicles into the presynaptic terminal membranes and increases the surface area, while endocytosis retrieves excess plasma membrane components followed by recycling to form other synaptic vesicles. Under normal conditions, the dynamics of balance between exocytosis and endocytosis are well preserved to maintain the correct surface area of the presynaptic terminal [32,33]. However, a mutant leucine-rich repeat kinase 2 (LRRK2) bacterial artificial chromosome (BAC) transgenic mouse model showed enlarged axonal endings in the striatal dopaminergic neurons, suggesting imbalance between exocytotic membrane addition and endocytic retrieval [34]. Excessive accumulation of presynaptic vesicles and enlargement of the VGLUT1-positive nerve terminals was also observed in *Snap25*^*S187A/S187A*^ mice [19]. Taking into consideration the synaptic vesicle cycle, our findings suggest that the balance of the cycle is likely biased toward decreased endocytosis.

The enlarged VGLUT1-positive nerve terminals of *Snap25*^*S187A/S187A*^ mice showed concomitant accumulation of αSyn and p-αSyn [19]. Kramer and Schulz-Schaeffer [5] have previously reported that 90% or even more of αSyn aggregates in DLB cases were located at the presynapses in the form of very small deposits. In parallel, dendritic spines were retracted, whereas the presynapses were relatively preserved, suggesting that neurotransmitter deprivation may explain the cognitive impairment in DLB [5,6]. While the presynaptic aggregates did not contain much p-αSyn in their examination [5,6], widespread varicosities and dot-like structures containing p-αSyn are commonly observed in αSyn-transgenic mouse model and human DLB brains [35,36]. This may represent axonal transport defects and presynaptic dysfunctions [35,36]. Recent study showed that mutant αSyn (A53T) diminished levels of various motor proteins in neurons [37], supporting this scenario. Alternatively, excessive amount of misfolded αSyn and p-αSyn may aggregate at synapses, physically preventing the targeting of other presynaptic proteins [5]. In experiments using *Caenorhabditis elegans* overexpressing human αSyn, four genes related to the endocytosis process were identified as genetic modifiers for αSyn toxicity [38]. They included two subunits of the adaptor protein (AP) complex 2, which interacts with clathrin and promotes presynaptic clathrin-mediated vesicle recycling [39]. Furthermore, proteomics analysis revealed that p-αSyn also preferentially interacted with the proteins involved in endocytosis, including clathrin heavy chain and subunit of AP-2 and AP-1 complexes, over the non-phosphorylated αSyn [40]. Clathrin-mediated recycling of exocytosed synaptic vesicles occurs in the periactive zone, a region adjacent to the active zone where synaptic vesicle is endocytosed [33]. Similarly, in *Snap25*^*S187A/S187A*^ mice, immunoelectron microscopy showed preferential localization of αSyn at the periactive zone of excitatory presynaptic nerve terminals. This might reflect the interaction of αSyn and p-αSyn with the proteins involved in clathrin-mediated endocytosis. Taking these findings together, presynaptic accumulation of αSyn and p-αSyn could disturb the endocytosis process and consequently contribute to the development of VGLUT1-positive terminal enlargement [19].

### Presynaptic accumulation of αSyn

Presynaptic accumulation of αSyn is considered an early event in the pathogenesis of α-synucleinopathies [4-6]. Mice overexpressing human αSyn showed presynaptic accumulation of αSyn and low DA release in the striatum. Stoica et al. [41] reported a “dying back” type of neuronal alteration, progressing from the dendrites to the axon and then to the perikaryon and nucleus in a spontaneously inherited autosomal recessive rat model for PD that overexpressed αSyn in mesencephalic area. Transmission electron microscopy (TEM) examination revealed that the retrograde pathological process in substantia nigra and striatum starts at the synaptic level by marked presynaptic accumulation of αSyn followed by post-synaptic degeneration of axonal terminals, dendrites and spine alterative changes and perikaryal aggregation of mitochondria with relative preservation of neuronal nuclei. These findings were associated with abnormal distribution of SNARE proteins, which colocalized with αSyn aggregates. Similarly, accumulation of SNARE proteins and αSyn were reported in the striatum of PD patients [18]. These observations suggest that SNARE dysfunction likely occurs at an early stage of pathogenesis in nigrostriatal dysfunction observed in PD. Considering the findings observed in the VGLUT1-positive nerve terminals, we expected that SNARE dysfunction might have induced presynaptic accumulation of αSyn, which consequently result in the development of neurodegenerative changes in the nigrostriatal system. However, contrary to our expectation, *Snap25*^*S187A/S187A*^ mice showed no significant neurodegenerative changes in nigrostriatal dopaminergic neurons, suggesting that SNARE dysfunction alone was insufficient to cause nigrostriatal degeneration as observed in PD, and appeared to be a downstream event associated with abnormal accumulation of αSyn.

## Conclusion

In conclusion, SNARE dysfunction leads to accumulation of endogenous αSyn in the corticostriatal nerve terminals. Presynaptic accumulation of αSyn is considered to be an early key event in the pathogenesis of α-synucleinopathies. Although the “prion-like” propagation hypothesis of αSyn, including tau and TAR DNA-binding protein 43 kDa, is currently receiving considerable attention worldwide, our findings provide an insight to understanding of the possible mechanisms that lead to presynaptic accumulation of endogenous αSyn. Moreover, given that SNAP-25 is reduced in the striatum of MSA brains [42], we speculate that a discontinuous pattern of αSyn pathologies usually found in MSA, *i.e.* glial cytoplasmic inclusions (GCIs) in the putaminal oligodendrocytes, and neuronal cytoplasmic inclusions (NCIs) and neuronal nuclear inclusions (NNIs) in the cortex [43,44], might be potentially linked through the presynaptic accumulation of αSyn in the corticostriatal neurons. Further investigations on the *Snap25* mutant mice with genetic ablation of αSyn would contribute to understanding the essential role of redistributed αSyn.

## Competing interest

The authors have no conflict of interest.

## Authors’ contribution

TY, YN, and CJC wrote the paper. HM supervised the work. All authors read and approved the final manuscript.

## References

[B1] Spillantini MG Goedert M The alpha-synucleinopathies: Parkinson’s disease, dementia with Lewy bodies, and multiple system atrophy Ann N Y Acad Sci 2000 920 16 27 11193145 10.1111/j.1749-6632.2000.tb06900.x

[B2] Galvin JE Lee VM Trojanowski JQ Synucleinopathies: clinical and pathological implications Arch Neurol 2001 58 186 190 10.1001/archneur.58.2.186 11176955

[B3] Yasuda T Nakata Y Mochizuki H Alpha-synuclein and neuronal cell death Mol Neurobiol 2013 47 2 466 483 10.1007/s12035-012-8327-0 22936307 PMC3589663

[B4] Neumann M Kahle PJ Giasson BI Ozmen L Borroni E Spooren W Muller V Odoy S Fujiwara H Hasegawa M Iwatsubo T Trojanowski JQ Kretzschmar HA Haass C Misfolded proteinase K-resistant hyperphosphorylated alpha-synuclein in aged transgenic mice with locomotor deterioration and in human alpha-synucleinopathies J Clin Invest 2002 110 1429 1439 12438441 10.1172/JCI15777 PMC151810

[B5] Kramer ML Schulz-Schaeffer WJ Presynaptic alpha-synuclein aggregates, not Lewy bodies, cause neurodegeneration in dementia with Lewy bodies J Neurosci 2007 27 1405 1410 10.1523/JNEUROSCI.4564-06.2007 17287515 PMC6673583

[B6] Schulz-Schaeffer WJ The synaptic pathology of alpha-synuclein aggregation in dementia with Lewy bodies, Parkinson’s disease and Parkinson’s disease dementia Acta Neuropathol 2010 120 131 143 10.1007/s00401-010-0711-0 20563819 PMC2892607

[B7] Maroteaux L Campanelli JT Scheller RH Synuclein: a neuron-specific protein localized to the nucleus and presynaptic nerve terminal J Neurosci 1988 8 2804 2815 3411354 10.1523/JNEUROSCI.08-08-02804.1988 PMC6569395

[B8] Iwai A Masliah E Yoshimoto M Ge N Flanagan L de Silva HA Kittel A Saitoh T The precursor protein of non-A beta component of Alzheimer’s disease amyloid is a presynaptic protein of the central nervous system Neuron 1995 14 467 475 10.1016/0896-6273(95)90302-X 7857654

[B9] Chandra S Gallardo G Fernandez-Chacon R Schluter OM Südhof TC Alpha-synuclein cooperates with CSPalpha in preventing neurodegeneration Cell 2005 123 383 396 10.1016/j.cell.2005.09.028 16269331

[B10] Burre J Sharma M Tsetsenis T Buchman V Etherton MR Südhof TC Alpha-synuclein promotes SNARE-complex assembly in vivo and in vitro Science 2010 329 1663 1667 10.1126/science.1195227 20798282 PMC3235365

[B11] Darios F Ruiperez V Lopez I Villanueva J Gutierrez LM Davletov B Alpha-synuclein sequesters arachidonic acid to modulate SNARE-mediated exocytosis EMBO Rep 2010 11 528 533 10.1038/embor.2010.66 20489724 PMC2897113

[B12] Murphy DD Rueter SM Trojanowski JQ Lee VM Synucleins are developmentally expressed, and alpha-synuclein regulates the size of the presynaptic vesicular pool in primary hippocampal neurons J Neurosci 2000 20 3214 3220 10777786 10.1523/JNEUROSCI.20-09-03214.2000 PMC6773130

[B13] Cabin DE Shimazu K Murphy D Cole NB Gottschalk W McIlwain KL Orrison B Chen A Ellis CE Paylor R Lu B Nussbaum RL Synaptic vesicle depletion correlates with attenuated synaptic responses to prolonged repetitive stimulation in mice lacking alpha-synuclein J Neurosci 2002 22 8797 8807 12388586 10.1523/JNEUROSCI.22-20-08797.2002 PMC6757677

[B14] Larsen KE Schmitz Y Troyer MD Mosharov E Dietrich P Quazi AZ Savalle M Nemani V Chaudhry FA Edwards RH Stefanis L Sulzer D Alpha-synuclein overexpression in PC12 and chromaffin cells impairs catecholamine release by interfering with a late step in exocytosis J Neurosci 2006 26 11915 11922 10.1523/JNEUROSCI.3821-06.2006 17108165 PMC6674868

[B15] Nemani VM Lu W Berge V Nakamura K Onoa B Lee MK Chaudhry FA Nicoll RA Edwards RH Increased expression of alpha-synuclein reduces neurotransmitter release by inhibiting synaptic vesicle reclustering after endocytosis Neuron 2010 65 66 79 10.1016/j.neuron.2009.12.023 20152114 PMC3119527

[B16] Südhof TC The synaptic vesicle cycle Annu Rev Neurosci 2004 27 509 547 10.1146/annurev.neuro.26.041002.131412 15217342

[B17] Sharma M Burre J Südhof TC CSPalpha promotes SNARE-complex assembly by chaperoning SNAP-25 during synaptic activity Nat Cell Biol 2011 13 30 39 10.1038/ncb2131 21151134

[B18] Garcia-Reitböck P Anichtchik O Bellucci A Iovino M Ballini C Fineberg E Ghetti B Della Corte L Spano P Tofaris GK Goedert M Spillantini MG SNARE protein redistribution and synaptic failure in a transgenic mouse model of Parkinson’s disease Brain 2010 133 2032 2044 10.1093/brain/awq132 20534649 PMC2892942

[B19] Nakata Y Yasuda T Fukaya M Yamamori S Itakura M Nihira T Hayakawa H Kawanami A Kataoka M Nagai M Sakagami H Takahashi M Mizuno Y Mochizuki H Accumulation of alpha-synuclein triggered by presynaptic dysfunction J Neurosci 2012 32 17186 17196 10.1523/JNEUROSCI.2220-12.2012 23197711 PMC6621870

[B20] Kataoka M Yamamori S Suzuki E Watanabe S Sato T Miyaoka H Azuma S Ikegami S Kuwahara R Suzuki-Migishima R Nakahara Y Nihonmatsu I Inokuchi K Katoh-Fukui Y Yokoyama M Takahashi M A single amino acid mutation in SNAP-25 induces anxiety-related behavior in mouse PLoS One 2011 6 e25158 10.1371/journal.pone.0025158 21949876 PMC3176821

[B21] Majewski H Iannazzo L Protein kinase C: a physiological mediator of enhanced transmitter output Prog Neurobiol 1998 55 463 475 10.1016/S0301-0082(98)00017-3 9670214

[B22] Morgan A Burgoyne RD Barclay JW Craig TJ Prescott GR Ciufo LF Evans GJ Graham ME Regulation of exocytosis by protein kinase C Biochem Soc Trans 2005 33 1341 1344 10.1042/BST20051341 16246114

[B23] Nagy G Matti U Nehring RB Binz T Rettig J Neher E Sorensen JB Protein kinase C-dependent phosphorylation of synaptosome-associated protein of 25 kDa at Ser187 potentiates vesicle recruitment J Neurosci 2002 22 9278 9286 12417653 10.1523/JNEUROSCI.22-21-09278.2002 PMC6758053

[B24] Orimo S Uchihara T Nakamura A Mori F Kakita A Wakabayashi K Takahashi H Axonal alpha-synuclein aggregates herald centripetal degeneration of cardiac sympathetic nerve in Parkinson’s disease Brain 2008 131 642 650 10.1093/brain/awm302 18079166

[B25] Gureviciene I Gurevicius K Tanila H Role of alpha-synuclein in synaptic glutamate release Neurobiol Dis 2007 28 83 89 10.1016/j.nbd.2007.06.016 17689254

[B26] Bamford NS Robinson S Palmiter RD Joyce JA Moore C Meshul CK Dopamine modulates release from corticostriatal terminals J Neurosci 2004 24 9541 9552 10.1523/JNEUROSCI.2891-04.2004 15509741 PMC6730145

[B27] Wickens JR Arbuthnott GW Dunnett SB, Bentivoglio M, Björklund A, Hökfelt T Structural and functional interactions in the striatum at the receptor level Dopamine, vol. 21 of Handbook of Chemical Neuroanatomy 2005 Amsterdam: Elsevier 199 236

[B28] Shimazaki Y Nishiki T Omori A Sekiguchi M Kamata Y Kozaki S Takahashi M Phosphorylation of 25-kDa synaptosome-associated protein. Possible involvement in protein kinase C-mediated regulation of neurotransmitter release J Biol Chem 1996 271 14548 14553 10.1074/jbc.271.24.14548 8662851

[B29] Iwasaki S Kataoka M Sekiguchi M Shimazaki Y Sato K Takahashi M Two distinct mechanisms underlie the stimulation of neurotransmitter release by phorbol esters in clonal rat pheochromocytoma PC12 cells J Biochem 2000 128 407 414 10.1093/oxfordjournals.jbchem.a022768 10965039

[B30] Shoji-Kasai Y Itakura M Kataoka M Yamamori S Takahashi M Protein kinase C-mediated translocation of secretory vesicles to plasma membrane and enhancement of neurotransmitter release from PC12 cells Eur J Neurosci 2002 15 1390 1394 10.1046/j.1460-9568.2002.01972.x 11994133

[B31] Burgoyne RD Morgan A Chaperoning the SNAREs: a role in preventing neurodegeneration? Nat Cell Biol 2011 13 8 9 10.1038/ncb0111-8 21173802

[B32] Hayes NVL Baines AJ Lee AG Smallsynapticvesicles Biomembranes: a multi-volume treatise 1996 Greenwich, CT: JAI 75 122

[B33] Haucke V Neher E Sigrist SJ Protein scaffolds in the coupling of synaptic exocytosis and endocytosis Nat Rev Neurosci 2011 12 127 138 10.1038/nrn2948 21304549

[B34] Li Y Liu W Oo TF Wang L Tang Y Jackson-Lewis V Zhou C Geghman K Bogdanov M Przedborski S Beal MF Burke RE Li C Mutant LRRK2(R1441G) BAC transgenic mice recapitulate cardinal features of Parkinson’s disease Nat Neurosci 2009 12 7 826 828 10.1038/nn.2349 19503083 PMC2845930

[B35] Saito Y Kawashima A Ruberu NN Fujiwara H Koyama S Sawabe M Arai T Nagura H Yamanouchi H Hasegawa M Iwatsubo T Murayama S Accumulation of phosphorylated alpha-synuclein in aging human brain J Neuropathol Exp Neurol 2003 62 644 654 12834109 10.1093/jnen/62.6.644

[B36] Scott DA Tabarean I Tang Y Cartier A Masliah E Roy S A pathologic cascade leading to synaptic dysfunction in alpha-synuclein-induced neurodegeneration J Neurosci 2010 30 8083 8095 10.1523/JNEUROSCI.1091-10.2010 20554859 PMC2901533

[B37] Chung CY Koprich JB Siddiqi H Isacson O Dynamic changes in presynaptic and axonal transport proteins combined with striatal neuroinflammation precede dopaminergic neuronal loss in a rat model of AAV alpha-synucleinopathy J Neurosci 2009 29 3365 3373 10.1523/JNEUROSCI.5427-08.2009 19295143 PMC2693917

[B38] Kuwahara T Koyama A Koyama S Yoshina S Ren CH Kato T Mitani S Iwatsubo T A systematic RNAi screen reveals involvement of endocytic pathway in neuronal dysfunction in alpha-synuclein transgenic C. elegans Hum Mol Genet 2008 17 2997 3009 10.1093/hmg/ddn198 18617532

[B39] Morgan JR Prasad K Hao W Augustine GJ Lafer EM A conserved clathrin assembly motif essential for synaptic vesicle endocytosis J Neurosci 2000 20 8667 8676 11102472 10.1523/JNEUROSCI.20-23-08667.2000 PMC6773056

[B40] McFarland MA Ellis CE Markey SP Nussbaum RL Proteomics analysis identifies phosphorylation-dependent alpha-synuclein protein interactions Mol Cell Proteomics 2008 7 2123 2137 10.1074/mcp.M800116-MCP200 18614564 PMC2577212

[B41] Stoica G Lungu G Bjorklund NL Taglialatela G Zhang X Chiu V Hill HH Schenk JO Murray I Potential role of α-synuclein in neurodegeneration: studies in a rat animal model J Neurochem 2012 122 4 812 822 10.1111/j.1471-4159.2012.07805.x 22639889

[B42] Tong J Wong H Guttman M Ang LC Forno LS Shimadzu M Rajput AH Muenter MD Kish SJ Hornykiewicz O Furukawa Y Brain alpha-synuclein accumulation in multiple system atrophy, Parkinson’s disease and progressive supranuclear palsy: a comparative investigation Brain 2010 133 172 188 10.1093/brain/awp282 19903734

[B43] Yoshida M Multiple system atrophy: α-synuclein and neuronal degeneration Neuropathology 2007 27 484 493 10.1111/j.1440-1789.2007.00841.x 18018485

[B44] Ubhi K Low P Masliah E Multiple system atrophy: a clinical and neuropathological perspective Trends Neurosci 2011 34 581 590 10.1016/j.tins.2011.08.003 21962754 PMC3200496

